# Monitoring circulating dipeptidyl peptidase 3 (DPP3) predicts improvement of organ failure and survival in sepsis: a prospective observational multinational study

**DOI:** 10.1186/s13054-021-03471-2

**Published:** 2021-02-15

**Authors:** Alice Blet, Benjamin Deniau, Karine Santos, Dirk P. T. van Lier, Feriel Azibani, Xavier Wittebole, Benjamin G. Chousterman, Etienne Gayat, Oliver Hartmann, Joachim Struck, Andreas Bergmann, Massimo Antonelli, Albertus Beishuizen, Jean-Michel Constantin, Charles Damoisel, Nicolas Deye, Salvatore Di Somma, Thierry Dugernier, Bruno François, Stephane Gaudry, Vincent Huberlant, Jean-Baptiste Lascarrou, Gernot Marx, Emmanuelle Mercier, Haikel Oueslati, Peter Pickkers, Romain Sonneville, Matthieu Legrand, Pierre-François Laterre, Alexandre Mebazaa, AdrenOSS-1 Study Investigators, Laterre, Dugernier, Huberlant, Marx, Hohn, Meier-Hellmann, Jaschinski, Kortgen, Francois, Lascarrou, Mercier, Desachy, Lasocki, Mebazaa, Mebazaa, Jacob, Gaudry, Pottecher, Constantin, Sonneville, Disomma, Antonelli, Beishuizen, Pickkers

**Affiliations:** 1 Department of Anesthesiology, Critical Care and Burn Center, Lariboisière - Saint-Louis Hospitals, DMU Parabol, AP–HP Nord, University of Paris, Paris, France; 2 Inserm UMR-S 942, Cardiovascular Markers in Stress Conditions (MASCOT), University of Paris, 2 rue Ambroise Paré, 75010 Paris, France; 3 University of Ottawa Heart Institute and University of Ottawa, Ottawa, ON Canada; 4 4TEEN4 Pharmaceuticals GmbH, Hennigsdorf, Germany; 5 Department of Intensive Care Medicine, Radboud University Medical Center, Geert Grooteplein Zuid 10, 6500 HB Nijmegen, The Netherlands; 6 Radboud Center for Infectious Diseases, Radboud University Medical Center, Nijmegen, The Netherlands; 7 Department of Critical Care Medicine, St Luc University Hospital, Université Catholique de Louvain, Brussels, Belgium; 8 SphingoTec GmbH, Hennigsdorf, Germany; 9 Department of Anesthesiology and Intensive Care Medicine, Fondazione Policlinico Universitario A. Gemelli IRCCS, Rome, Italy; 10 Department of Intensive Care, Medische Spectrum Twente, Enschede, The Netherlands; 11 GRC 29, AP-HP, DMU DREAM, Department of Anaesthesiology and Critical Care, Pitié-Salpêtrière Hospital, Sorbonne University, Paris, France; 12 Department of Medical and Toxicological Critical Care, Lariboisière Hospital, Federation of Toxicology APHP, Paris-Diderot University, Paris, France; 13 Sant’ Andrea Hospital, Rome, Italy; 14 Clinique St Pierre, Ottignies, Belgium; 15 ICU Department, CHU Dupuytren, Limoges, France; 16 INSERM CIC 1435/UMR 1092, Limoges, France; 17 Hôpital Louis Mourier, Colombes, France; 18 Hôpital Jolimont, Haine-St-Paul, Belgium; 19 Centre Hospitalier Universitaire de Nantes, Nantes, France; 20 Klinik Für Operative Intensivmedizin Und Intermediate Care, Universitätsklinikum Der RWTH, Aachen, Germany; 21 CHU de Tours, Tours, France; 22 Hopital Bichat Claude-Bernard, Paris, France; 23 Department of Anesthesia and Perioperative Care, University of California San Francisco, San Francisco, USA; 24 Department of Critical Care Medicine, Saint Luc University Hospital, Université Catholique de Louvain, Avenue Hippocrate 10, 1200 Brussels, Belgium

**Keywords:** DPP3, Biomarker, Outcome, Sepsis, Septic shock, Organ dysfunction

## Abstract

**Background:**

Dipeptidyl peptidase 3 (DPP3) is a cytosolic enzyme involved in the degradation of various cardiovascular and endorphin mediators. High levels of circulating DPP3 (cDPP3) indicate a high risk of organ dysfunction and mortality in cardiogenic shock patients.

**Methods:**

The aim was to assess relationships between cDPP3 during the initial intensive care unit (ICU) stay and short-term outcome in the AdrenOSS-1, a prospective observational multinational study in twenty-four ICU centers in five countries. AdrenOSS-1 included 585 patients admitted to the ICU with severe sepsis or septic shock. The primary outcome was 28-day mortality. Secondary outcomes included organ failure as defined by the Sequential Organ Failure Assessment (SOFA) score, organ support with focus on vasopressor/inotropic use and need for renal replacement therapy. cDPP3 levels were measured upon admission and 24 h later.

**Results:**

Median [IQR] cDPP3 concentration upon admission was 26.5 [16.2–40.4] ng/mL. Initial SOFA score was 7 [5–10], and 28-day mortality was 22%. We found marked associations between cDPP3 upon ICU admission and 28-day mortality (unadjusted standardized HR 1.8 [CI 1.6–2.1]; adjusted HR 1.5 [CI 1.3–1.8]) and between cDPP3 levels and change in renal and liver SOFA score (*p* = 0.0077 and 0.0009, respectively). The higher the initial cDPP3 was, the greater the need for organ support and vasopressors upon admission; the longer the need for vasopressor(s), mechanical ventilation or RRT and the higher the need for fluid load (all *p* < 0.005). In patients with cDPP3 > 40.4 ng/mL upon admission, a decrease in cDPP3 below 40.4 ng/mL after 24 h was associated with an improvement of organ function at 48 h and better 28-day outcome. By contrast, persistently elevated cDPP3 at 24 h was associated with worsening organ function and high 28-day mortality.

**Conclusions:**

Admission levels and rapid changes in cDPP3 predict outcome during sepsis.

*Trial Registration* ClinicalTrials.gov, NCT02393781. Registered on March 19, 2015.

## Introduction

Circulating dipeptidyl peptidase 3 (cDPP3) is an ubiquitous, intracellular, peptidase involved in the degradation of various cardiovascular and endorphin mediators [1, 2]. When cell injury occurs, cDPP3 is released into the circulation. Prognostic properties of cDPP3 have already been demonstrated in critically ill patients suffering from cardiogenic shock and burn [3–5]. Apart from its function as a predictive biomarker of clinical outcome, we further showed, in pre-clinical studies, that cDPP3 exerts negative inotropic effects, working as a cardiac depressant factor [6]. These deleterious effects were reversed by Procizumab, a monoclonal anti-DPP3 neutralizing antibody [3]. This implies that cDPP3 may not only be a prognostic marker, but may also exert deleterious cardiovascular effects, representing a therapeutic target.

In a pilot study of septic patients, elevated cDPP3 levels upon ICU admission were observed in septic shock patients and in non-survivors [7]. In the present ancillary study from the Adrenomedullin and Outcome in Sepsis and Septic Shock 1 (AdrenOSS-1) study [8], we further investigated whether initial and repeated cDPP3 measurements during the early phase of sepsis may predict evolution of organ function and 28-day survival.

## Materials and methods

### Study design

AdrenOSS-1 was a prospective, observational study performed in 24 centers from five European countries (France, Belgium, The Netherlands, Italy and Germany) [8]. Patients (*n* = 585) were recruited from June 2015 to May 2016. The study protocol was approved by the local ethics committees and was conducted in accordance with Directive 2001/20/EC, as well as Good Clinical Practices (I.C.H. version 4 of May 1, 1996 and Decision of November 24, 2006) and the declaration of Helsinki. Criteria were previously described [8]. Briefly, main inclusion criteria was patients admitted to the ICU for severe sepsis or septic shock based on the former sepsis definition [9]. The primary endpoint was 28-day mortality. Secondary endpoints included organ failure (as defined by the SOFA score) and organ support, vasopressor/inotrope use, fluid balance and use of renal replacement therapy (RRT) and mechanical ventilation [10]. The use of organ support was left to the discretion of the clinician in charge of the patient in accordance with current practice. Acute kidney injury (AKI) was defined by KDIGO classification. Etiologies of AKI were not reported.

### Collection of patient data

Upon admission, demographics (age, sex), body mass index (BMI), origin of sepsis, presence of shock, type of ICU admission, organ dysfunction scores (Sequential Organ Failure Assessment, SOFA; Acute Physiologic Assessment and Chronic Health Evaluation II, APACHE II), pre-existing comorbidities (i.e. treated within the last year), past medical history, laboratory values, as well as organ support were recorded and research blood was collected for measurement of cDPP3 and other markers.

After patient enrolment, the following data were collected daily during the first week: SOFA score, antimicrobial therapies, fluid balance, ventilation status, Glasgow coma scale, central venous pressure, need for RRT, invasive procedures for sepsis control and vasopressor/inotrope treatment. Moreover, discharge status and mortality were recorded up to day 28 after ICU admission.

### Sample collection

Blood for the central laboratory was sampled within 24 h after ICU admission and 24 h after the first sample. Samples were subsequently processed and plasma stored at  − 80˚C before transfer to the central lab for blinded cDPP3 measurement organized by the study sponsor [7] (4TEEN4 Pharmaceuticals GmbH, Hennigsdorf, Germany).

### Statistical analyses

Results are presented as number (*n*) and percentage (%), or median and inter-quartile range (IQR), as appropriate. Group comparisons for continuous variables were performed using the Kruskal–Wallis test. Categorical data were compared using Pearson's Chi-squared Test for Count Data. Biomarker data were log-transformed, if necessary.

Cox proportional-hazards regression was used to analyse the effect of risk factors on survival in uni- and multivariable analyses. The assumptions of proportional hazard were tested for all variables. For continuous variables, hazard ratios (HR) were standardized to describe the HR for a biomarker change of one IQR. 95% confidence intervals (CI) for risk factors and significance levels for chi-square (Wald test) are given. The predictive value of each model was assessed by the model likelihood ratio chi-square statistic. The concordance index (C index) is given as an effect measure. It is equivalent to the concept of AUC adopted for binary outcome. For multivariable models, a bootstrap corrected version of the C index is given. To test for added predictive value, we used the likelihood ratio chi-square test for nested models to assess whether cDPP3 adds predictive value to a clinical model or a risk score. To assess the calibration accuracy of a multivariable Cox regression model, estimates of predicted survival probability will be plotted against observed survival in the data set. To this end, samples will be grouped into up to five groups according to their predicted survival, ensuring a minimum of 100 samples per group. Bootstrap corrected estimates are reported. Time-dependent Cox regression was used to evaluate the association of serial biomarker data and mortality. Survival curves plotted by the Kaplan–Meier method using quartiles of cDPP3 were used for illustrative purposes. Receiver-operating-characteristic (ROC) curves were constructed to illustrate the predictive performance for dichotomized endpoints. When dichotomizing the population based on cDPP3 was needed, the 3rd quartile was used (40.4 ng/mL), as the median (26.5 ng/mL) was within the normal range and therefore not a clinically meaningful cut point (upper normal range of DPP3 in plasma of healthy individuals is 41.2 ng/mL).

A two-sided p-value of 0.05 was considered statistically significant. All analyses were performed using R version 3.4.3 (http://www.r-project.org, library rms, Hmisc) and Statistical Package for the Social Sciences (SPSS) version 22.0 (SPSS Inc., Chicago, Illinois, USA).

## Results

Among the 585 patients included in AdrenOSS-1 study, 581 had a cDPP3 assessment. Admission demographics and details of clinical and biological parameters of the 581 septic patients are summarized in Table 1. Median [IQR] cDPP3 plasma levels upon admission in all Adren OSS-1 patients was 26.5 ng/mL [16.2–40.4].

**Table 1:** Admission demographic patient characteristics

Patient characteristics	All	Low cDPP3	High cDPP3	*p* value*	*n*
*Epidemiological data*	*n* = 581	*n* = 436	*n* = 145		
cDPP3 at admission (ng/mL)	26.5 [16.2–40.4]	19.8 [14.5–28.9]	67.7 [48.7–120.7]	–	
Age (year)	66 [55–75]	66 [56–76]	65 [53–75]	0.2498	
Male (no. %)	363 (62.5)	277 (63.5)	86 (59.3)	0.4176	
Body mass index (kg/m^2^)	25.7 [22.9–30.2]	25.5 [22.7–30.1]	26.1 [23.4–31.1]	0.1363	
Septic shock at admission (yes)	292 (50.3)	201 (46.1)	91 (62.8)	0.0007	
*Origin of sepsis*				0.0727	
Lung	218 (37.5)	151 (34.6)	67 (46.2)		
Blood stream	90 (15.5)	76 (17.4)	14 (9.7)		
Urinary tract	62 (10.7)	43 (9.9)	19 (13.1)		
Catheter	29 (5.0)	22 (5)	7 (4.8)		
Peritonitis	30 (5.2)	23 (5.3)	7 (4.8)		
Endocarditis	31 (5.3)	23 (5.3)	8 (5.5)		
Other	121 (20.8)	98 (22.5)	23 (15.9)		
Medical history**					
Any cardiac comorbidity (yes)	398 (68.5)	300 (68.8)	98 (67.6)	0.8642	
Chronic heart failure (yes)	59 (10.2)	38 (8.7)	21 (14.5)	0.0652	
Hypertension (yes)	292 (50.3)	220 (50.5)	72 (49.7)	1.0000	
Diabetes mellitus (yes)	159 (27.4)	122 (28)	37 (25.5)	0.6286	
Any non-cardiac comorbidity (yes)	413 (71.1)	308 (70.6)	105 (72.4)	0.7627	
Chronic renal disease (yes)	76 (13.1)	57 (13.1)	19 (13.1)	1.0000	
Active/recent malignant tumors (yes)	124 (21.3)	96 (22)	28 (19.3)	0.6090	
Smoking (active, yes)	116 (20)	88 (20.2)	28 (19.3)	0.9238	
COPD (yes)	89 (15.3)	70 (16.1)	19 (13.1)	0.5267	
Any chronic medication (yes)	370 (63.7)	281 (64.4)	89 (61.4)	0.5712	
Immunosuppressive therapy (yes)	46 (7.9)	32 (7.3)	14 (9.7)	0.4733	
*Physiological values at admission*					
Mean blood pressure (mmHg)	75 [64–90]	75 [65–90]	76 [62–90]	0.7372	
Heart rate (bpm)	104 [89–119]	101 [88–117]	108 [96–128]	0.0005	
Fluid balance (mL)	1930 [600–3556]	1800 [570–3270]	2398 [805–4731]	0.0059	
Urine output for 24 h (mL)	1000 [450–1900]	1130 [571–2000]	600 [202–1495]	< 0.0001	
PaO_2_/FiO_2_	228 [137–342]	237 [146–364]	191 [111–300]	0.0012	
*Laboratory values at admission*					
Lactate (mmol/L)	1.4 [1.0–2.2]	1.3 [0.9–2]	2.1 [1.2–3.48]	< 0.0001	*n* = 560
Arterial pH	7.38 [7.30–7.44]	7.4 [7.32–7.45]	7.33 [7.24–7.41]	< 0.0001	
Bilirubin (µmol/L)	11 [6–19]	10 [6–18]	12 [7–22]	0.1141	
Platelets (10^9^/L)	190 [121–274]	190 [123–273.75]	190 [112–269]	0.7341	
Creatinine (mg/dL)	1.35 [0.86–2.24]	1.26 [0.81–2.05]	1.71 [1.04–2.9]	0.0001	
BUN or urea (mg/dL)	61.3 [37.0–106.3]	58.3 [34.2–97]	72 [44–120.1]	0.0012	
Hematocrit (%)	34 [29–38]	34 [29–38]	35 [30–39]	0.0395	
White blood count (per mm3)	12,480 [7200–18560]	13,000 [7560–18600]	11,350 [6810–17040]	0.0958	
Troponin T (ng/mL)	42 [18–152]	38 [17–129]	54 [25–234]	0.0786	*n* = 152
PCT (ng/mL)	11.4 [1.9–49.8]	9.1 [1.6–37.4]	16.3 [3.6–70.0]	0.0194	*n* = 330
NT-proBNP (pg/mL)	4382 [1525–11565]	3339 [1294–7643]	9589 [4863–20107]	0.0003	*n* = 117
*Organ support at admission*					
Mechanical ventilation:				< 0.0001	
Invasive	217 (37.3)	134 (30.7)	83 (57.2)		
Non-invasive	131 (22.5)	98 (22.5)	33 (22.8)		
None	233 (40.1)	204 (46.8)	29 (20)		
Renal replacement therapy	49 (8.4)	19 (4.4)	30 (20.7)	< 0.0001	
Vasopressors/inotropes at admission	347 (59.7)	246 (56.4)	101 (69.7)	0.0066	
*Organ dysfunction scores*					
SOFA (points)	7 [5–10]	7 [4–9]	9 [5–12]	< 0.0001	*n* = 508
APACHE II (points)	15 [11–20]	15 [11–19]	18 [13–23]	< 0.0001	
*Length of stay (days)*					
ICU	5 [2–10]	5 [2–8]	6 [2–15]	0.0487	
*Outcome*					
AKI within 7 days	359 (61.8)	189 (56.6)	113 (77.9)	< 0.0001	
28-day, deaths (%)	126 (21.7)	66 (15.1)	60 (41.4)	< 0.0001	
90-day, deaths (%)	165 (28.4)	94 (21.6)	71 (49)	< 0.0001	

Comparisons were performed between patients with cDPP3 above (high cDPP3) and below (low cDPP3) 40.4 ng/mL (3rd quartile) upon admission. APACHE Acute Physiology and Chronic Health Evaluation, BNP Brain-derived natriuretic peptide, BUN Blood urea nitrogen, CNS Central nervous system, COPD Chronic obstructive pulmonary disease, cDPP3 circulating Dipeptidyl peptidase 3, ICU Intensive care unit, NT-proBNP N-terminal brain natriuretic peptide, PaO2/ FiO2 Ratio of partial pressure of arterial oxygen to fraction of inspired oxygen, PCT Procalcitonin, SOFA Sequential Organ Failure Assessment Among the *n* = 121 patients with origin of sepsis labelled as “other”, source of infection were: bile duct infection (*n* = 12), CNS (*n* = 4), skin and soft tissue (*n* = 10), gynaecologic (*n* = 2), “unknown” (*n* = 26), abdominal (*n* = 53), and other origins with less than 3 counts each (*n* = 14). *p Value from nonparametric Kruskal–Wallis or chi-square test, respectively. ** Most common comorbidities reported individually

We present the statistical results based on continuous data and used quartiles for categorization to visualize the results. High cDPP3 levels (defined by concentrations above the 3rd quartile (40.4 ng/mL, which also represents the DPP3 upper normal range in healthy individuals) measured upon admission were associated with worse metabolic parameters, worse renal and cardiac functions, higher SOFA score, longer ICU stay and higher 28 and 90 day-mortality in comparison to low cDPP3 values (Table 1). Of note, patients with septic shock (*n* = 292) had a significantly higher cDPP3 concentration upon admission than patients with severe sepsis (29.1 ng/mL [18.0–48.2] versus 23.2 ng/mL [15.2–35.1], *p* = 0.0001).

### cDPP3 levels upon ICU admission and mortality

Over the 28-day follow-up period, 126 patients (22%) died (33/289 (11%) of patients diagnosed with severe sepsis and 93/292 (32%) of patients diagnosed with septic shock). Admission characteristics of survivors versus non-survivors are shown in Additional file 8: Table 1. In univariate analysis, cDPP3 was associated with 28-day mortality (c index 0.692, standardized HR 1.8 [CI 1.6–2.1], *p* < 0.0001). For comparison, the c index for SOFA score was 0.729 (standardized HR 3.5 (2.6–4.6)) and for lactate 0.720 (standardized HR 1.8 (1.6–2.0)).

In a Cox proportional-hazard model adjusted for age, gender, comorbidities (cardiac or non-cardiac), diagnosis (severe sepsis, septic shock) and lactate, cDPP3 plasma levels upon admission remained independently associated with 28-day mortality (added chi-square 33.0, *p* < 0.0001, c index increase from 0.756 to 0.776, standardized HR 1.5 (1.3–1.8)). Additional file 1: Fig. 1 shows the calibration plot for the multivariable model. Overall, the model is well calibrated. A multivariate model further revealed that cDPP3 levels upon admission had incremental prognostic value on top of APACHE II or SOFA score (added chi-square 44.0 and 25.6, respectively, both *p* < 0.0001), as well as on top of plasma lactate (added chi-square 43.6, *p* < 0.0001) or procalcitonin (PCT, added chi-square 63.4, *p* < 0.0001), when used as a continuous variable.

**Fig. 1:**
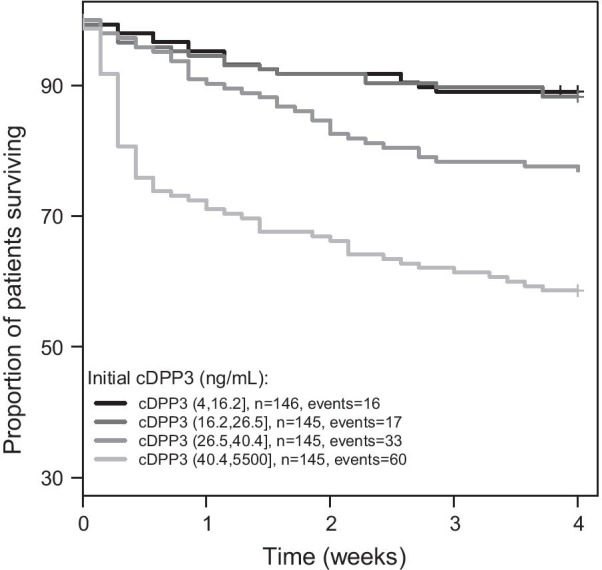
Twenty-eight-day Kaplan–Meier survival curves for quartiles of cDPP3 at admission. Standardized HR is 1.8 [CI 1.6–2.1], HR comparing patients with cDPP3 above and below 40.4 pg/mL (3rd quartile) is 3.4 [CI 2.4–4.8]

Kaplan–Meier analysis based on quartiles illustrates the association between cDPP3 plasma levels upon admission and 28-day mortality in all studied patients (Fig. 1). Notably, the survival rate for patients with cDPP3 levels above the 3rd quartile (40.4 ng/mL) sharply decreases within the first week (Fig. 1). Time-dependent AUC analysis of admission cDPP3 illustrates the superiority of cDPP3 compared to lactate and PCT for short-term mortality prediction (Additional file 3: Fig. 3). This remains true in subgroups of severe sepsis and septic shock (Additional file 4: Fig. 4a, b). Additional file 2: Fig. 2 shows the ROC curve for association between cDPP3 and 28-day mortality.

### cDPP3 levels upon admission and organ dysfunction

**Fig. 2:**
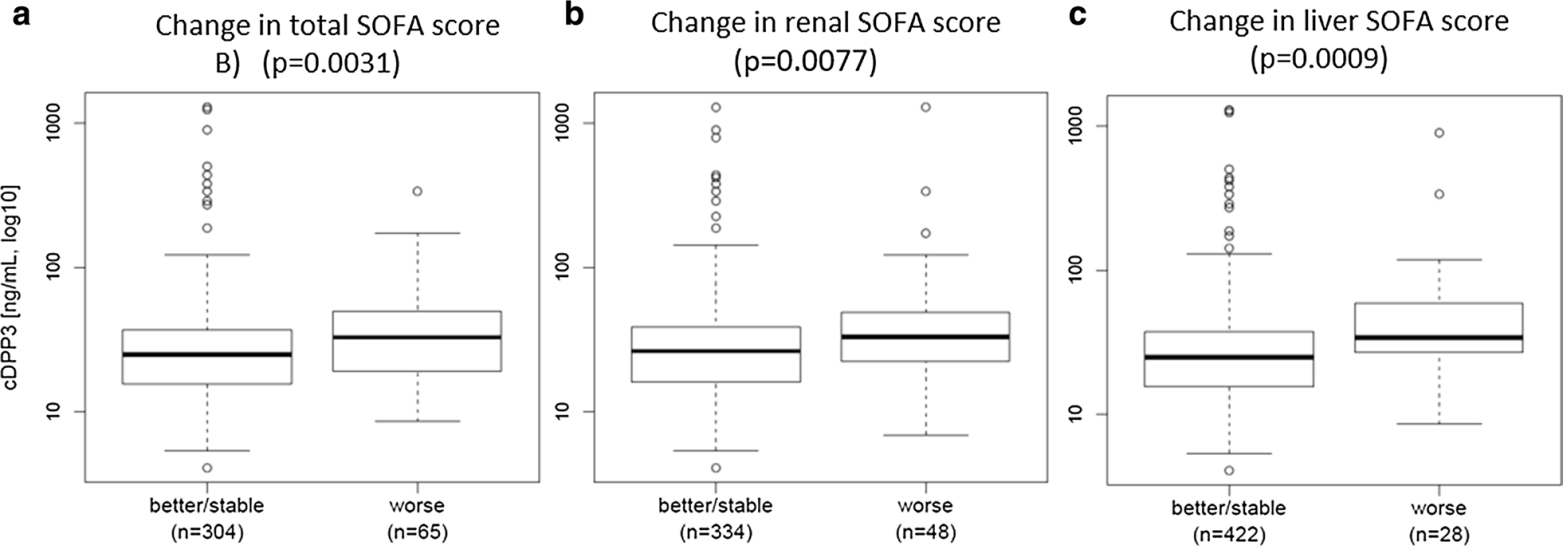
Association between cDPP3 levels at admission and 48 h change in **a** SOFA score (*p* = 0.0031), **b** renal SOFA score (*p* = 0.0009), and **c** liver SOFA score (*p* = 0.0009). Of note, this figure includes patients alive at 48 h and no missing data. In few cases, patients were discharged alive prior to 48 h: the 48 h SOFA was set at 0

cDPP3 levels upon admission correlated with the initial SOFA score (*p* < 0.0001) (Additional file 5: Fig. 5). In addition, initial cDPP3 plasma levels were high in patients that had worsening of their total, renal and liver SOFA score in the first 48 h of ICU stay (Fig. 2).

**Fig. 3:**
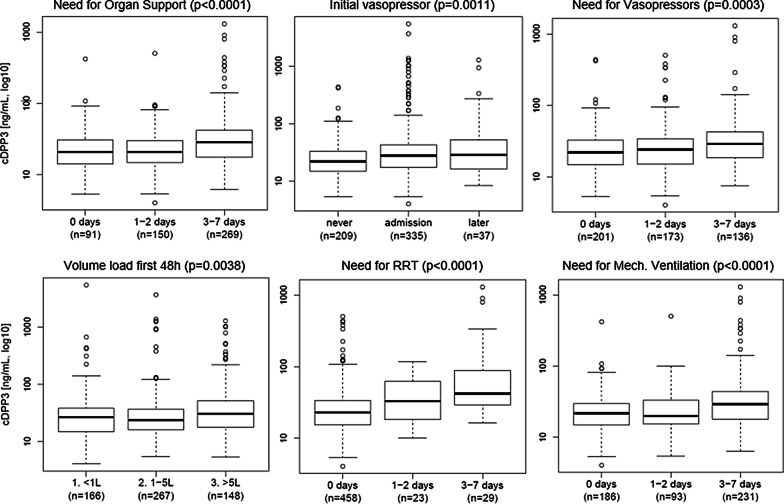
cDPP3 level at admission and the need for and duration of organ support in 7-day survivors. Organ support is defined as need for cardiac, renal or respiratory support. Cardiac support is defined as need for vasopressors, renal as need for RRT and respiratory support as need for mechanical ventilation

Figure 3 further indicates that initial cDPP3 levels were elevated in 7-day survivors with need for organ support, high demand of vasopressors upon admission, and prolonged vasopressor(s) need, as well as in patients that required mechanical ventilation, RRT and high fluid volume administration in the first week post admission. cDPP3 levels at admission were also significantly associated with need and duration of invasive mechanical ventilation (*p* > 0.0001) (Additional file 6: Fig. 6). Critically ill patients with high cDPP3 at admission were more likely to develop AKI within 7 days (Table 1). Further analyses showed greater cDPP3 levels in AKI versus non-AKI (*p* = 0.0001) (see Additional file 7: Fig. 7 for further details).

### Dynamic variation in cDPP3 levels and outcomes

**Fig. 4:**
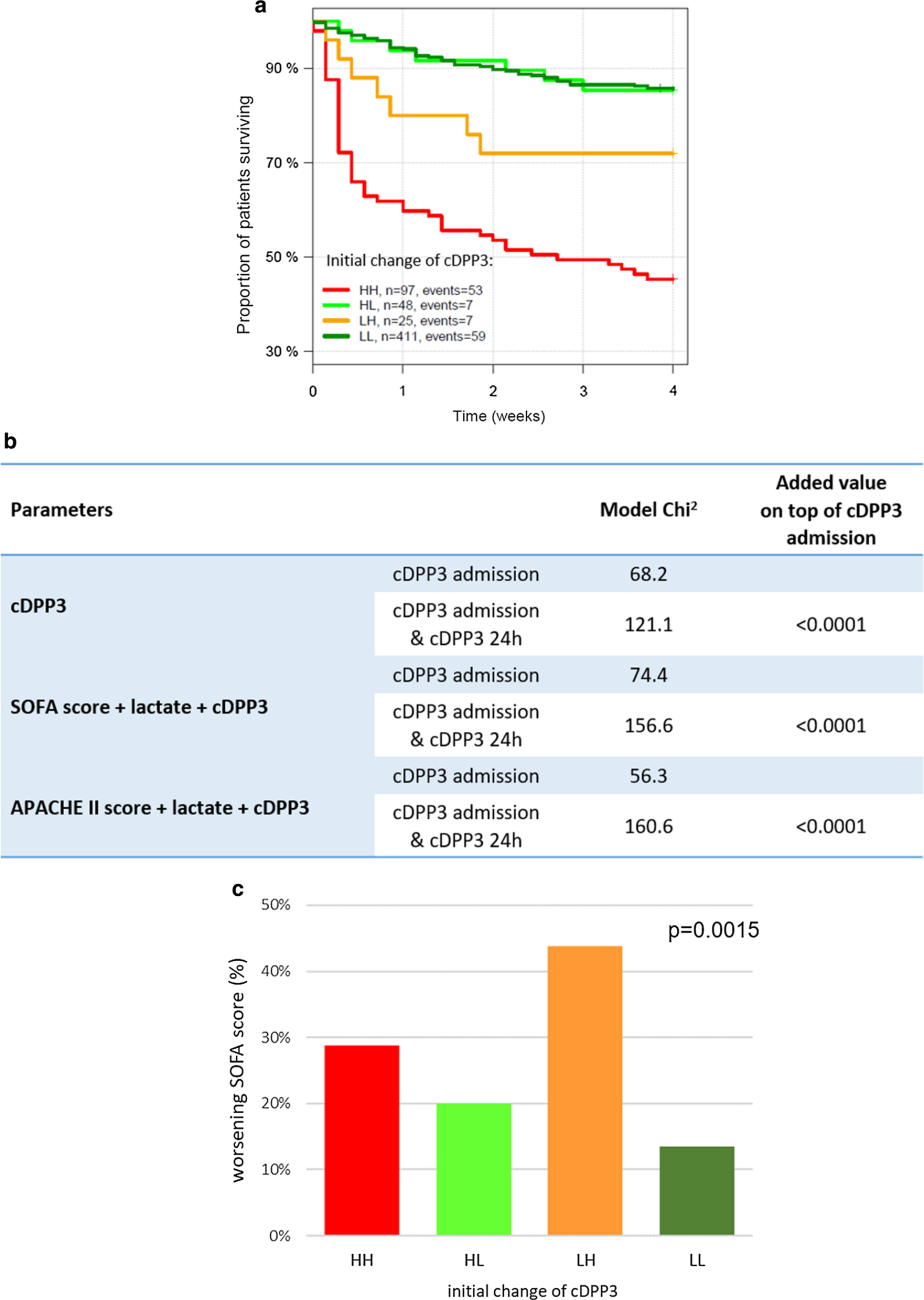
Association between the changes of circulating DPP3 (cDPP3) levels over 24 h and mortality (**A**) Association between cDPP3 and 28-day mortality in time-dependent Cox regression, and worsening of SOFA score within 48 h in patients alive at 48 h (**C**) HR between high-high (HH) (levels of cDPP3 remained high) and high-low (HL) (levels of cDPP3 declining over 24 h) 0.18 (95% CI 0.08–0.41). HR between low-low (LL) (levels of cDPP3 remaining low) and low–high (LH) (levels of cDPP3 increasing over 24 h) 2.2 [1.0–4.8], *p* = 0.0015). For patients discharged prior to 48 h, the 48 h SOFA was set to 0

Combining admission and 24 h cDPP3 measurements via time-dependent Cox regression shows that the 24 h cDPP3 measurement provides significant added value on top of the admission value (added Chi^2^ 52.9, *p* < 0.0001). The added value of 24 h cDPP3 remains an independent predictor of 28-day mortality after adjustment for SOFA score and lactate (added Chi^2^ 82.2, *p* < 0.0001) or APACHE II score and lactate (added Chi^2^ 104.3, *p* < 0.0001), with scores and lactate measured upon admission. For details see Fig. 4b. Figure 4a shows that changes in the concentrations of cDPP3 during the first 24 h were associated with different outcomes. Patients were divided into four groups based on admission and 24 h cDPP3 concentrations using the cut-off value of 40.4 ng/mL: remaining low (low-low, LL), admission high to low at 24 h (HL), admission low to high at 24 h (LH), and remaining high (high-high, HH). Figure 4a shows that patients with dropping cDPP3 values from high levels upon admission to low levels after 24 h (HL) had reduced 28-day mortality risk compared to patients in whom cDPP3 levels remained high after 24 h (HH) (HR 0.18 [0.08–0.41]). More importantly, the HL-patients had a similar risk of mortality compared to patients who remained low (LL) (HR 1.1 [0.52–2.4]). On the other hand, patients in whom cDPP3 became high after 24 h (LH) displayed an increased mortality risk compared to patients who started low and remained low (LL) (HR 2.2 [CI 1.0–4.8]). Regarding organ function, regardless of the levels of cDPP3 upon admission, high concentrations of cDPP3 levels 24 h later (LH and HH) were associated with worsening of total SOFA score within 48 h, as well as AKI (Fig. 4c and Additional file 7: Fig. 7c).

## Discussion

This ancillary analysis of AdrenOSS-1 study, a prospective ICU multinational observational cohort study on biomarkers in sepsis, revealed that high cDPP3 levels upon admission had marked prognostic value and was associated with impaired short-term outcome. More importantly, early reduction of cDPP3 levels towards normal values was associated with improved organ function and 28-day survival, while persistent elevation of DPP3 predicted impaired outcome more accurately than current disease severity scores and e.g. lactate. Our study confirms previous work [7] but now in a larger cohort of ICU patients admitted with sepsis that cDPP3 upon admission is higher in septic shock compared to severely septic patients.

Our study further showed that patients admitted to the ICU with high cDPP3 levels, defined by values above the 3^rd^ quartile (40 ng/mL), had threefold greater risk of 28-day death. cDPP3 levels at admission were also elevated in patients with a worsening of total SOFA score, as well as worsening of renal and liver SOFA sub scores at 48 h. In addition, high cDPP3 plasma levels were significantly associated with increased need for organ support, namely cardiovascular, mechanical ventilation and/or renal replacement therapy in 7-day survivors. These results indicate that high cDPP3 plasma levels are associated with multiple organ injury and reflect patient severity. Our study also indicates that cDPP3 upon admission better predicts short-term mortality in sepsis than lactate or PCT. The marked prognostic value of cDPP3 for short-term mortality has been previously described in two cohorts of cardiogenic shock patients [3, 4]. In cardiogenic shock patients, levels of cDPP3 upon admission were higher than in septic shock patients and the threshold associated with higher short-term mortality was approx. 60 ng/ml compared to 40 ng/ml in septic patients [4]. Altogether, our study indicates that cDPP3 upon admission may be a promising prognostic factor in critically ill patients.

In addition to the prognostic value of cDPP3 upon admission, dynamics of cDPP3 during the initial phase were informative as well. We described marked association between low cDPP3 (≤ 40.4 ng/mL) at 24 h and low 28-day mortality, even in patients with initial high cDPP3 levels upon admission. Of interest, low cDPP3 at 24 h was also associated with improvement of organ function at day 7. Moreover, our study showed that prognostic properties of serial measurements of cDPP3 (admission and 24 h) remained strong even when adjusted to ICU risk scores and lactate. Altogether, these findings indicate that cDPP3 measured once, or even better twice, in the first 24 h may markedly guide intensivists in the early management of septic patients.

Higher concentrations of cDPP3 were associated with more pronounced cardiovascular dysfunction, such as need for vasopressor therapy. Of interest, recent pre-clinical studies showed that the modulation of cDPP3 by specific antibodies may improve hemodynamic [3]. This implies that, apart from being a biomarker of cell damage, cDPP3 may exert functional properties and that treatment with an antibody may be beneficial in patients with elevated cDPP3 levels. Future studies should explore whether those novel therapies might improve organ function and survival in septic patients with high cDPP3.

Limitations of our study include its observational nature. The prognosis value of DPP3 as a biomarker will need to be confirmed in further studies. AdrenOSS-1 study was designed before the sepsis-3 definition. Therefore, the definition of severe sepsis and septic shock in AdrenOSS-1 was based on the “sepsis-2” definition [9]. It is, however, likely that cDPP3 plasma levels could have demonstrated similar prognostic properties in septic patients included with the sepsis-3 definition [11], especially as we show that cDPP3 has additional predictive properties over lactate. Furthermore, our data did not record parameters of myocardial function. We are, therefore, unable to link detrimental prognostic properties of cDPP3 to negative effects on heart contraction, as demonstrated in animals [3]. While we did show that higher cDDP3 concentrations are associated with greater need for hemodynamic support, further studies are clearly needed to fully clarify the impact of cDPP3 on cardiac contractility. In addition, our data did not record the indication for renal replacement therapy (RRT), neither the etiologies of AKI. Strengths of this study include its international conduct, indicating that the observed associations are generalizable and its considerable sample size, strengthening the point estimates of our findings.

## Conclusion

In this ancillary analysis of AdrenOSS-1, we showed a strong association between high levels of cDPP3 in plasma upon ICU admission and clinical outcome, both short and mid-term. More importantly, normalization of cDPP3 levels in the first days after ICU admission was associated with marked improvement of total SOFA score and with lower 28-day mortality suggesting the need for cDDP3 monitoring during hospitalization. Combined with preclinical data demonstrating beneficial effects of DPP3 inhibition, these results warrant further investigation of the therapeutic potential to modulate the cDPP3 pathway in septic shock patients.

## Supplementary information

**Additional file 1**: Figure 1. Calibration plot designed for the multivariable model including age, gender, comorbidities (cardiac or non-cardiac), diagnosis (severe sepsis, septic shock), lactate and cDPP3 plasma levels upon admission for predicting 28-day mortality.

**Additional file 2**: Figure 2. ROC curve for association between cDPP3 and 28-day mortality. Based on the cut point 40.4 ng/mL (Q3) sensitivity was 47.6% and specificity 81.3%.

**Additional file 3**: Figure 3. Time-dependent AUC plot for all-cause mortality for cDPP3, PCT and lactate, up to 28-days follow up. cDPP3 elicits the strongest prognostic properties in the first days.

**Additional file 4**:Figure 4A and B. Twenty-eight-day Kaplan-Meier survival curves for cDPP3 on admission, based on cDPP3 quartiles from the full population, for severe sepsis (A) and septic shock patients (B).

**Additional file 5**: Figure 5. Association of DPP3 and SOFA score at baseline (p<0.0001).

**Additional file 6**: Figure 6. cDPP3 level at admission and the need for and duration of respiratory support, defined as invasive mechanical ventilation only (p<0.0001).

**Additional file 7**: Figure 7A, B and C. (A) Box plot of cDPP3 level at admission upon AKI and non AKI patients. (B) ROC curve for association between cDPP3 and AKI. (C) Association between the changes of circulating DPP3 (cDPP3) levels over 48h and AKI.

**Additional file 8**: Table.

## Data Availability

Data used for this study are available on request.

## Abbreviations

APACHE II: Acute Physiologic Assessment and Chronic Health Evaluation II
AUC: Area Under Curve
BMI: Body Mass Index
cDPP3: Circulating Dipeptidyl Peptidase 3
CI: 95% Confidence intervals
DPP3: Dipeptidyl Peptidase 3
HR: Hazard Ratios
ICU: Intensive Care Unit
IQR: Inter-Quartile Range
RRT: Renal Replacement Therapy
SOFA: Sequential Organ Failure Assessment
