# Supplementary figures and images for: Monitoring circulating dipeptidyl peptidase 3 (DPP3) predicts improvement of organ failure and survival in sepsis: a prospective observational multinational study
Source: Crit Care. 2021 Feb 15;25:61. doi: 10.1186/s13054-021-03471-2 (PMC7885215; doi:10.1186/s13054-021-03471-2)

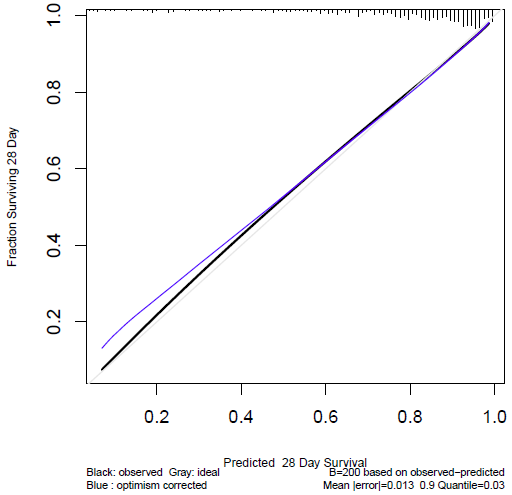

Supplement: Supplementary file 1 — Additional file 1: Figure 1. Calibration plot designed for the multivariable model including age, gender, comorbidities (cardiac or non-cardiac), diagnosis (severe sepsis, septic shock), lactate and cDPP3 plasma levels upon admission for predicting 28-day mortality. [file 13054_2021_3471_MOESM1_ESM.png]

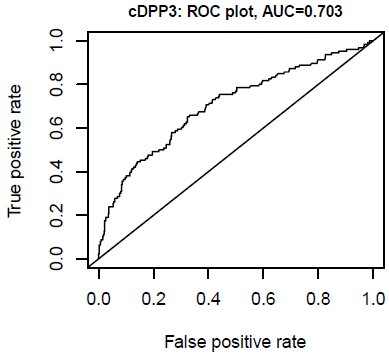

Supplement: Supplementary file 2 — Additional file 2: Figure 2. ROC curve for association between cDPP3 and 28-day mortality. Based on the cut point 40.4 ng/mL (Q3) sensitivity was 47.6% and specificity 81.3%. [file 13054_2021_3471_MOESM2_ESM.png]

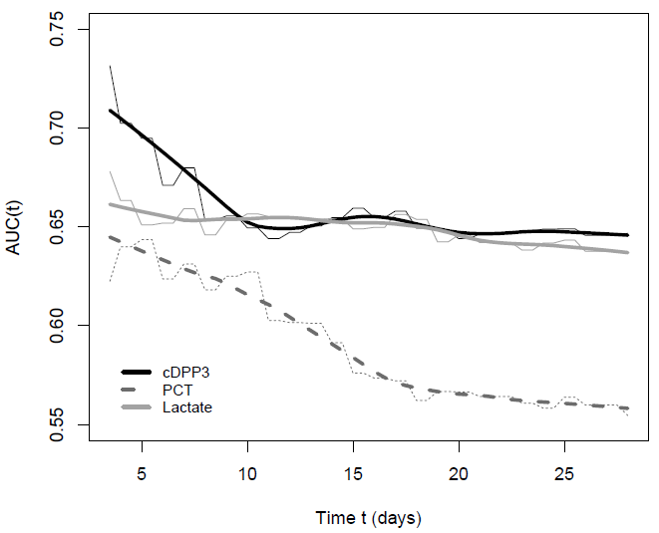

Supplement: Supplementary file 3 — Additional file 3: Figure 3. Time-dependent AUC plot for all-cause mortality for cDPP3, PCT and lactate, up to 28-days follow up. cDPP3 elicits the strongest prognostic properties in the first days. [file 13054_2021_3471_MOESM3_ESM.png]

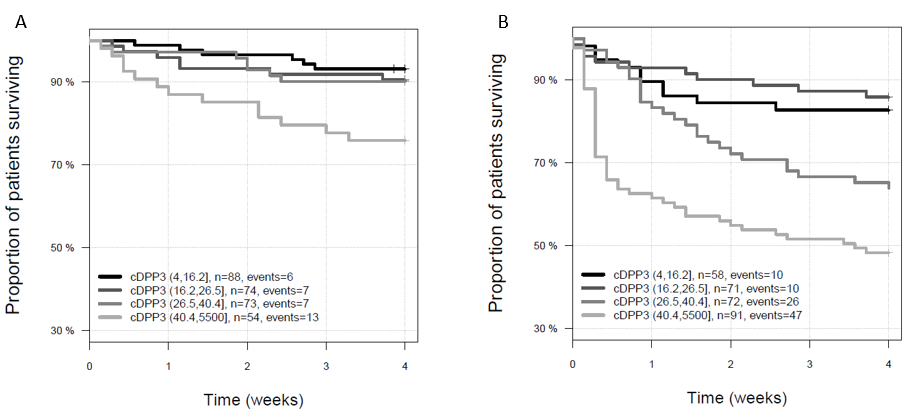

Supplement: Supplementary file 4 — Additional file 4:Figure 4A and B. Twenty-eight-day Kaplan-Meier survival curves for cDPP3 on admission, based on cDPP3 quartiles from the full population, for severe sepsis (A) and septic shock patients (B). [file 13054_2021_3471_MOESM4_ESM.png]

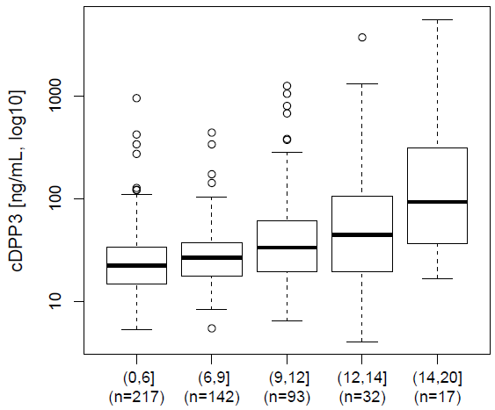

Supplement: Supplementary file 5 — Additional file 5: Figure 5. Association of DPP3 and SOFA score at baseline (p<0.0001). [file 13054_2021_3471_MOESM5_ESM.png]

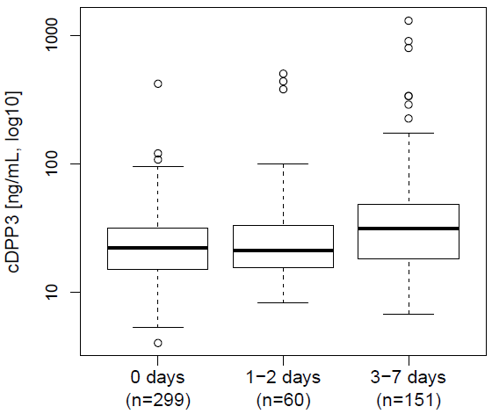

Supplement: Supplementary file 6 — Additional file 6: Figure 6. cDPP3 level at admission and the need for and duration of respiratory support, defined as invasive mechanical ventilation only (p<0.0001). [file 13054_2021_3471_MOESM6_ESM.png]

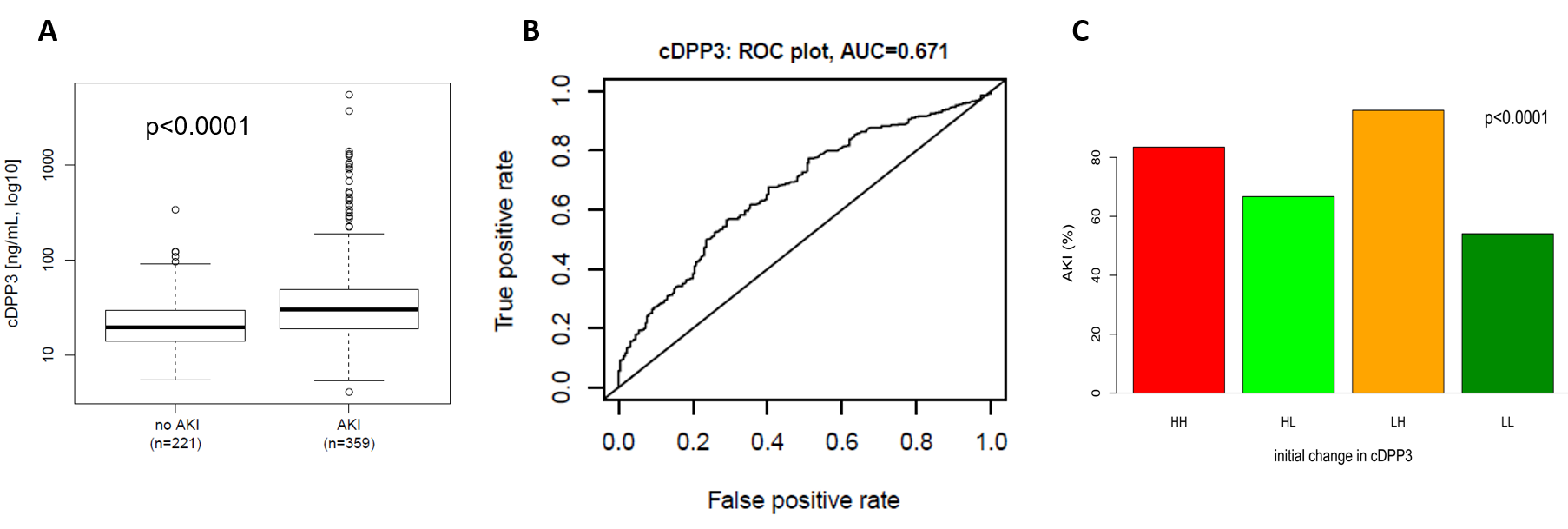

Supplement: Supplementary file 7 — Additional file 7: Figure 7A, B and C. (A) Box plot of cDPP3 level at admission upon AKI and non AKI patients. (B) ROC curve for association between cDPP3 and AKI. (C) Association between the changes of circulating DPP3 (cDPP3) levels over 48h and AKI. [file 13054_2021_3471_MOESM7_ESM.png]
