# Supplementary material for: Monitoring circulating dipeptidyl peptidase 3 (DPP3) predicts improvement of organ failure and survival in sepsis: a prospective observational multinational study
Source: Crit Care. 2021 Feb 15;25:61. doi: 10.1186/s13054-021-03471-2 (PMC7885215; doi:10.1186/s13054-021-03471-2)
Supplement: Supplementary file 8 — Additional file 8: Table. [file 13054_2021_3471_MOESM8_ESM.docx]

**Supplemental table 1. Admission demographic patient characteristics of 28-day survivors and non-survivors**

| **Patient characteristics** | **28-day survivors** | **28-day non-survivors** | **p-value*** | **n** |
| --- | --- | --- | --- | --- |
| **Epidemiological data** | **n=455** | **n=126** |  |  |
| cDPP3 at admission (ng/mL) | 23.2 [15.4-35.1] | 36.8 [26.3-85.8] | <0.0001 |  |
| Age (year) | 65 [54-74] | 70 [63-78] | 0.0002 |  |
| Males (No. %) | 277 (60.9) | 86 (68.3) | 0.1588 |  |
| Body Mass Index (kg/m²) | 25.71 [22.46-30.28] | 25.52 [23.68-29.83] | 0.5623 |  |
| Septic shock at admission (yes) | 199 (43.7) | 93 (73.8) | <0.0001 |  |
| **Origin of sepsis** |  |  | 0.0184 |  |
| Lung | 176 (38.7) | 42 (33.3) |  |  |
| Blood stream | 81 (17.8) | 9 (7.1) |  |  |
| Urinary tract | 43 (9.5) | 19 (15.1) |  |  |
| Catheter | 23 (5.1) | 6 (4.8) |  |  |
| Peritonitis | 23 (5.1) | 7 (5.6) |  |  |
| Endocarditis | 21 (4.6) | 10 (7.9) |  |  |
| Other | 88 (19.3) | 33 (26.2) |  |  |
|  |  |  |  |  |
| **Medical history**** |  |  |  |  |
| Any cardiac comorbidity (yes) | 297 (65.3) | 101 (80.2) | 0.0021 |  |
| Chronic Heart Failure (yes) | 42 (9.2) | 17 (13.5) | 0.3420 |  |
| Hypertension (yes) | 213 (46.8) | 79 (62.7) | 0.0035 |  |
| Diabetes Mellitus (yes) | 119 (26.2) | 40 (31.7) | 0.4071 |  |
| Any non-cardiac comorbidity (yes) | 309 (67.9) | 104 (82.5) | 0.0020 |  |
| Chronic renal disease (yes) | 57 (12.5) | 19 (15.1) | 0.5059 |  |
| Active/recent malignant tumors (yes) | 88 (19.3) | 36 (28.6) | 0.0115 |  |
| Smoking (active, yes) | 90 (19.8) | 26 (20.6) | 0.9676 |  |
| COPD (yes) | 67 (14.7) | 22 (17.5) | 0.1193 |  |
| Any chronic mediaction (yes) | 277 (60.9) | 93 (73.8) | 0.0103 |  |
| Immunosuppressive therapy (yes) | 37 (8.1) | 9 (7.1) | 0.8592 |  |
| **Physiological values at admission** |  |  |  |  |
| Mean blood pressure (mmHg) | 76 [65-90.75] | 73 [60-90] | 0.1686 |  |
| Heart rate (bpm) | 103 [88-117] | 109 [93.25-130] | 0.0076 |  |
| Fluid Balance (mL) | 1749 [500-3142.75] | 2860 [1205-5042] | <0.0001 |  |
| Urine output for 24 hours (mL) | 1170 [581-2000] | 550 [241.25-1445] | <0.0001 |  |
| PaO2/FiO2 | 234 [146-354] | 192 [114-299.75] | 0.0070 |  |
| **Laboratory values at admission** |  |  |  |  |
| Lactate (mmol/L) | 1.23 [0.9-1.9] | 2.2 [1.36-4.05] | <0.0001 | n=560 |
| Arterial pH | 7.4 [7.32-7.45] | 7.33 [7.27-7.4] | <0.0001 |  |
| Bilirubin (umol/L) | 10 [6-18] | 13 [6.75-22.5] | 0.1199 |  |
| Platelets (10^9^/L) | 192 [124-280] | 165.5 [95.25-260] | 0.0257 |  |
| Creatinine (mg/dL) | 1.29 [0.8-2.14] | 1.56 [1.11-2.6] | 0.0028 |  |
| BUN or Urea (mg/dL) | 57.06 [34.23-101.45] | 74.14 [49.07-119.84] | 0.0003 |  |
| Hematocrit (%) | 34 [30-38] | 33 [28-39] | 0.3682 |  |
| White blood count (per mm3) | 13320 [8000-18900] | 10330 [4150-16890] | 0.0007 |  |
| Troponin T, maximum on day 1 | 33 [18-131] | 56 [31-226.5] | 0.1342 | n=152 |
| PCT, maximum on day 1 (ng/mL) | 10.7 [1.91-49.39] | 11.82 [2.15-52.52] | 0.6690 | n=330 |
| NT-proBNP, maximum on day 1 | 3400 [1027-9052] | 7249 [4563-25007] | 0.0007 | n=117 |
| **Organ support at admission** |  |  |  |  |
| Mechanical ventilation: |  |  | <0.0001 |  |
| Invasive | 140 (30.8) | 77 (61.1) |  |  |
| Non-invasive | 107 (23.5) | 24 (19) |  |  |
| None | 208 (45.7) | 25 (19.8) |  |  |
| Renal replacement therapy | 27 (5.9) | 22 (17.5) | 0.0002 |  |
| Vasopressors/inotropes at admission | 245 (53.8) | 102 (81) | <0.0001 |  |
| **Organ dysfunction scores** |  |  |  |  |
| SOFA (points) | 6 [4-9] | 10 [7.5-12] | <0.0001 | n=508 |
| APACHE II (points) | 15 [10-18.5] | 19 [16-23] | <0.0001 |  |
| **Length of stay (days)** |  |  |  |  |
| ICU | 5 [2.25-10] | 4 [2-8] | 0.0122 |  |
| **Mortality (%)** |  |  |  |  |
| 28-day, deaths | 0 (0) | 126 (100) | - |  |
| 90-day, deaths | 39 (8.6) | 126 (100) | - |  |

*Abbreviations:* APACHE Acute Physiology and Chronic Health Evaluation, BNP Brain-derived natriuretic peptide, BUN Blood urea nitrogen, CNS Central nervous system, COPD Chronic obstructive pulmonary disease, cDPP3 circulating Dipeptidyl peptidase 3, ICU Intensive care unit, NT-proBNP N-terminal brain natriuretic peptide, PaO2/ FiO2 Ratio of partial pressure of arterial oxygen to fraction of inspired oxygen, PCT Procalcitonin, SOFA Sequential Organ Failure Assessment * p Value from nonparametric Kruskal-Wallis or chi-square test, respectively a Most common comorbidities reported individually
